# The not-for-profit form and translational research: Kerr revisited?

**DOI:** 10.1186/1479-5876-3-19

**Published:** 2005-04-29

**Authors:** Keith A Joiner

**Affiliations:** 1 University of Arizona College of Medicine, Office of the Dean, University of Arizona Health Sciences Center, 1501 N. Campbell Avenue, Room 2205, Tucson, Arizona, 85724, USA

## Abstract

Translational research conducted in academic health centers is confounded by the organizational structure in which the work is performed. Investigators must obtain research funding and appropriate recognition as a part of a research team in a not-for-profit environment which has more readily rewarded basic work, and individual accomplishments. What results is a unique form of conflict of interest, best understood by relating the basic principles underlying the not-for-profit form to the conduct of translational research in the AHC setting.

## Introduction

In his classic article entitled "On the Folly of Rewarding A while Hoping for B", Kerr outlines a common phenomenon in organizational management and behavior [1]. Employees receive explicit or implicit incentives (rewards) to carry out one set of activities (A), while the organization hopes that the same employees will also or instead carry out a different set of activities (B). Conflict of interest, not of the conventional type [2], is created between what is rewarded and what is desired. This situation often helps to explain the dichotomy between desired and actual results. Kerr cites teaching in higher educational settings as a prime example. Faculty are expected by the organization to be devoted and excellent teachers, yet are rewarded for endeavors other than teaching.

The premise of this article is that the same dichotomy operates for translational research in AHC. The dichotomy emanates largely from the fact that AHC translational research is typically conducted in the not-for-profit (NPO) setting [3-7]. The dearth of physician-scientists working at the translational interface is one consequence.

### Forces Promoting the Not-for-Profit Form

In her book, "Strategic Management for Nonprofit Organizations", Sharon Oster suggests three forces which promote the existence of the not-for-profit form [3]. A paramount consideration is contract failure. Many if not most NPO produce goods or services which are difficult to evaluate in terms of quality. Under these circumstances, it is both problematic and inadvisable to write or enforce a contract between the provider and the stakeholders/consumer/buyer. Instead, provision of services must be based on trust between these two entities. This is the most direct manifestation of public trust. A second force promoting NPO is public failure, typically meaning that the government cannot or will not provide the service, even though the service is considered a public good. The last feature is worker sorting. Individuals are attracted to work for NPO based on the altruistic mission of the organization. Employees often serve as volunteers or at compensation levels below the market, if compared with for profit enterprises.

These three forces are inextricably linked to the most commonly appreciated feature of NPO: the non-distribution constraint, which in turn is linked to the special tax status. The non-distribution constraint states that "any financial surplus generated by operations cannot be distributed to those in control of the operation", which includes the stakeholders (in theory the public) and the employees of the NPO. While this does not preclude NPO from having positive cash flows, increasing net assets, or growing balances in endowment accounts, funds from these accounts cannot be proportionately distributed in the form of bonuses, or other direct benefits.

To what extent do non-profit Academic Health Centers (AHC) conform to these criteria and to what extent do they diverge? Answers to this question vary for the clinical, educational, and research missions. This article will not address the former two areas, and will approach only a subset of the latter – basic and disease-oriented research (as defined by Goldstein and Brown [8]), with a particular focus on translational research. Patient-oriented research, and specifically therapeutic trials, are best considered under other paradigms.

### Mission Statements and Research in AHC

How can the goal of the research mission within AHC best be summarized? For most NPO, the mission statement represents the most succinct and important expression of the mission. A review of web sites for more than 100 AHC revealed that only 1 in 5 had readily accessible and explicit institutional mission statements. From these mission statements, the statement referable to research was evaluated. More explicit vision statements accompanied the mission statements in some cases. A nearly constant theme was that research should be translated into advantage for human health. While this informal survey suggests that AHC should be more attentive to explicit statements of mission and vision, it also demonstrates the expected and hoped for mandate that translational investigation be the goal of research in the not-for-profit AHC setting.

### Translational Research and Contract Failure in AHC

If the research mission of AHC is to make basic discoveries which are translated into advantage for maintaining or improving human health, does contract failure exist for the translational research mission? Stakeholders (the public) cannot easily judge the quality of scientific research conducted by individual investigators, nor can they readily determine the likelihood that advances will be translated into new diagnostics, therapeutics or preventative strategies. By these criteria, the conditions for contract failure are met.

Given the existence of contract failure, to what extent does the non-profit form provide reassurances that individual investigators will neither provide a sub-standard product, nor work towards goals which are not mission-based.? With regard to the first issue, the guarantees for quality are strict, since investigators are scrutinized by their peers for the quality of the research conducted.

With regard to the second issue, the answer is much more ambiguous when considering research funded through the National Institutes of Health or other federal sources (Department of Veteran's Affairs, Department of Defense). There is a widely recognized tendency for review groups to fund well written applications for which the hypothesis being addressed is important, the preliminary data are compelling, but most importantly, the proposed experiments are likely to be successful and can be completed in the time frame of the award (personal observation). Direct applicability to human health may be discussed, but rarely is used as an essential criteria in the funding decision. Highly creative approaches, which the investigator believes provide the best chance for making a real impact for human health, will typically not be funded without substantial preliminary data to indicate that they will be successful. The complexities of conducting translational research, including insuring sufficient access to patients and volunteers, meeting regulatory challenges, conducting research in an only partially controlled setting, and dealing with expensive infrastructure, all preclude preparation of applications which have the crispness and apparent sophistication of more basic proposals. Similarly, extremely applied experiments, which the scientist believes will most rapidly translate into an advantage for human health, are often poorly received because they are not considered sufficiently innovative. (Of note, in the for-profit arena, these latter constraints are minimized, given the imperative to establish marketable products). While funding agencies and AHC are vocal advocates of translational research, reward systems are structured to support less risky basic activities. Kerr is at work.

These contentions cannot be readily proved or disproved, because there is no set of uniform criteria by which to make these judgments. The most relevant data comes from comparison of NIH funding success rates for clinical vs. non-clinical research. Two analyses [9,10], spanning an era (1997–2004) where substantial emphasis was placed on funding clinical research, revealed substantially higher success rates for non-clinical than for clinical applications. More general perspectives reach the same conclusion [11-13] Some of the above assertions are recognized by the National Institutes of Health (NIH). As stated in the description of high risk research in the NIH Roadmap , "the NIH peer-review process is oriented to fund so-called 'low-risk' proposals that advance well-established areas of science. This leaves many more speculative, or 'high-risk', proposals without an obvious mechanism of NIH support". One proposed solution is the Directors Pioneer award . The Pioneer award provides up to $500,000 per year of direct costs for 5 years for investigators to explore ideas that are considered risky at their inception, yet for which strong preliminary data is lacking.

In contrast to the mandate for funding the Pioneer award, the usually unstated "logic" of review groups for more common funding mechanisms (e.g. R01) is different. The assumption is made that allocating funds towards "safer" projects, even when quite basic and without direct connection to human disease, is ultimately the best way to accomplish the mission, since it is probable that those discoveries will ultimately contribute directly or indirectly to more applied solutions.

Irrespective of whether this "logic" is or can ultimately be validated, the premise of this manuscript is that it creates a conflict of interest for translationally-oriented investigators. This conflict is typified in published papers and in applications for research funding. It is commonplace for the introduction and discussion/conclusion of grant applications and manuscripts to make reference to the relevance of the work for advancing human health. While often done quite tangentially, this addresses the mission of central importance to the public and arguably to the AHC. If the author were to state explicitly that the likelihood was extremely low that the proposed or completed research would ultimately have any significant relevance to human health (or would even be cited by other authors), the application or manuscript would be seriously compromised. In contrast, throughout the remainder of the application or manuscript (and hence in the work itself), the investigator is compelled to focus on a "mission" which is different – convincing fellow scientists that the work is valid, irrespective of its relationship to the broader mission of importance to the public. If instead, the requirement at each step of the work was to make the connection to human disease, the form of the experiments would be often need to be altered in a fashion precluding the rigorous proof expected of high level scientific investigation.

In the for-profit arena, by contrast, a focus on product development with direct applicability to human health necessarily dominates the investigative and development process. Funding decisions are made internally. Preparation of peer-reviewed manuscripts is not an expected end-goal of much of the investigation. Research form follows desired organizational outcome more directly.

### Translational Research and Public Failure in AHC

Does public failure exist for the translational research mission of AHC?

Leaving aside the legitimate debate as to whether advancements in biomedical research in AHC are public goods, it is a reasonable assumption that government cannot or will not directly provide the vast array of biomedical advances desired by the public. What is and what should be the linkage between government and NPO in addressing this public failure? NPO often serve as a mechanism for delivery of public services, in partnership with the government. In these situations (for example education, transportation, environment), the government establishes the services to be provided, and contracts with NPO to provide those services.

Is this model applicable to biomedical research? In some respects yes, but in many respects no. By far the largest provider of funds for biomedical research in the United States is the federal government, through the National Institutes of Health. Who determines the criteria for providing the funds to the investigators, and what is the role of government in insuring that there is an applied outlet for the work [14]? The substantive scientific conditions, which are the dominant consideration in determining the likelihood of funding success, are established by the investigators themselves, in the form of study sections and review groups. These panels typically consist of investigators who have obtained NIH funding, usually in a somewhat related area, and who will generally apply for additional NIH grant support in the future. In other words, the grant is effectively awarded by the investigators for the investigators, a notion far quite far astray from the concept of public failure. No matter how supportive the investigators may be of the government's overarching goal, it is both human nature and part of one's training to support "good science" (the kind of science they consider themselves to do and to be rewarded for) over other factors. While the administrative conditions for the award are set by the NIH, while budget allocations to individual institutes or for particular initiatives do influence the scope of the funded research, and while NIH program officers have some discretion in insuring that grant awards fulfill program goals, it is not even clear that budget allocations faithfully match the burden of disease or government intent [15-17].

These considerations are not meant to call into question the rigor or validity of the review process, both of which are held in high regard. Similarly, it remains the case that the public recognizes the value of basic research, temporizing the extent of "public failure". Nonetheless, this perspective indicates that the hope for more and better translational research is not faithfully aligned with the reward systems for most investigators. Based on the considerations discussed above with regard to contract failure, it illustrates the potential conflict of interest between the government and the public on the one hand, and the government and the academic research community on the other [14,18,19].

### Translational Research and Worker Sorting in AHC

The pure concept of worker sorting suggests that biomedical investigators work in AHC based on the organizational mission, and at some personal sacrifice, at least financially. Is this formulation largely correct for the translational research enterprise? Only partially.

Investigators wishing to establish an independent career in research will nearly always opt to do so in AHC. The attraction of doing so is not primarily the mission of the organization, but rather the freedom of choice and the breadth of opportunities which are provided to excel. This is particularly true for bona fide translational research, which is most easily conducted in the environment of an AHC. Such opportunities, particularly for more junior investigators, are simply not present in for-profit research enterprises. In AHC, investigators strive to gain both recognition and support, for purposes of career advancement and career stability. If such individuals respond to a mission statement at all, it is likely to be to the professional organization/s of which they are members or occasionally to a much broader mission statement which transcends the institution. Investigators can even be likened to mini-religious organizations, with a fervent zeal for their own research which dominates other considerations [20]. The institutional mission and organizational structure of the AHC may even conflict with the individual mission statement of the investigator, the latter of which often promotes and condone individual pursuits but may or may not be aligned with those of the organization [21]. At the same time, the rewards to the investigator for conducting translational research transcend some of these considerations, and include improved patient recruitment, with attendant increases in patient volume and clinical income, increased personal recognition, and funds from licensing agreements for intellectual property.

Programmatic and team-based investigation is increasingly recognized as the most rapid and efficient structure for scientific progress for many if not most questions – i.e. to accomplish the research mission. In the for-profit-setting, rewards can be readily distributed based on the performance of the organization, and/or of individual teams within the organization. In AHC, academic promotion, research funding and professional recognition are far more readily achieved from an individual standpoint. Once again leaving aside the cogent arguments supporting both sides of this dilemma, individual accomplishment is most readily rewarded, while funding agencies, scientific organizations, AHC and the public hope that team-based research will predominate.

There is a growing body of literature describing the impediments to interdisciplinary research (IDR) in AHC, and suggesting strategies to remove those impediments [22]. Based on a survey of over 300 respondents, the five most important recommendations for institutions to facilitate IDR were a.) fostering a collaborative environment, b.) providing faculty incentives and organizing hiring policies and tenure processes to support IDR, c.) providing seed funding, d.) creating cross-department budgeting mechanisms, and e.) generating strategic plans that promote IDR.

Of importance, NIH Roadmap initiatives have established a series of awards which facilitate interdisciplinary research . These new awards provide funding for training of scientists in interdisciplinary strategies, for creation of interdisciplinary centers, and for conferences which catalyze collaboration between the life and physical sciences. In turn, AHC must respond by reconfiguring the reward system (promotions, professional recognition) to accurately value team-based research. Otherwise a new manifestation of conflict of interest will result.

This requires new strategies to appropriately identify team members, to evaluate their contributions to the team, and to adequately reward them for these contributions. While detailed lists of recommendations [22] are beyond the scope of this manuscript, selected budgetary policies are reflective of the philosophy: a.) crediting a percentage of indirect costs from all projects to support cross departmental infrastructure for IDR, b.) providing seed money, staff and space to support IDR, c.) creating a campus-wide inventory of equipment to enhance sharing of across facilities.

## Conclusion

The "misalignments" described here derive from genuine uncertainties about the best approaches to meeting the research mission in AHC. They also emanate from a reward system in AHC (and more broadly in universities) which recognizes and rewards individual accomplishments more than group performance. These conflicts are not necessarily unhealthy, and can be described as balancing of competing priorities.

New initiatives outlined in the NIH Roadmap favor funding for programmatic, team-based research. Concurrently, AHC must configure the reward system to appropriately and accurately value contributions of team members. Doing so will minimize conflict of interest, and will allow AHC to more faithfully meet their mission.

## List of Abbreviations

AHC, academic health center; NIH, national institutes of health; NPO, not-for-profit organization

## Competing interests

The author(s) declare that they have no competing interests.
